# Acute Graft-Versus-Host Disease After Humanized Anti-CD19-CAR T Therapy in Relapsed B-ALL Patients After Allogeneic Hematopoietic Stem Cell Transplant

**DOI:** 10.3389/fonc.2020.573822

**Published:** 2020-09-29

**Authors:** Pengjiang Liu, Meijing Liu, Cuicui Lyu, Wenyi Lu, Rui Cui, Jia Wang, Qing Li, Nan Mou, Qi Deng, Donglin Yang

**Affiliations:** ^1^Department of Hematology, Tianjin First Central Hospital, Tianjin, China; ^2^The First Central Clinical College of Tianjin Medical University, Tianjin, China; ^3^Shanghai Genbase Biotechnology Co., Ltd., Tianjin, China; ^4^State Key Laboratory of Experimental Hematology, National Clinical Research Center for Blood Diseases, Institute of Hematology and Blood Diseases Hospital, Chinese Academy of Medical Sciences and Peking Union Medical College, Tianjin, China

**Keywords:** acute lymphoblastic leukemia, relapse, allogeneic hematopoietic stem cell transplantation, chimeric antigen receptor (CARs), graft-versus-host disease, cytokine release syndrome

## Abstract

We studied the acute graft-versus-host disease (GVHD) after humanized anti-CD19-CAR T therapy in relapsed B-acute lymphoblastic leukemia (ALL) patients after allogeneic hematopoietic stem cell transplant (allo-HSCT). Fifteen B-ALL patients were enrolled in our study. Thirteen patients (86.67%) achieved a complete response (CR) or CR with incomplete count recovery. The donor chimerism of the 13 patients reached 99.86 ± 0.21%. The development of aGVHD was observed in 10 patients (66.67%). Six patients developed grade I-II of aGVHD, while the other four patients developed grade III-IV of aGVHD. The notable adverse events were grade 1–2 cytokine release syndrome (CRS) in 10 patients and grade 3–4 CRS in five patients. Two patients died of infection, while another patient died of sudden cardiac arrest. The anti-CD19-CAR T cells were not eliminated in peripheral blood when the patients developed aGVHD. However, we did not observe their expansion peaks again in the process of aGVHD. During the aGVHD, the peaks of IL-6 and TNF-a were correlated with aGVHD levels. By May 31, 2020, the rates of leukemia-free survival (LFS) and overall survival (OS) at 180 days were 53.846 and 61.638%, respectively. All the patients who survived to date experienced aGVHD after humanized anti-CD19-CAR T cell therapy.

**Trial registration:** The patients were enrolled in clinical trials of *ChiCTR-ONN-16009862* and *ChiCTR1800019622*.

## Introduction

Allogeneic hematopoietic stem cell transplant (allo-HSCT) is an effective therapy for B-cell acute lymphoblastic leukemia (B-ALL). However, some patients relapse after allo-HSCT with a median survival of fewer than 6 months (1). Thus, treating these patients remains a challenge. Therapies for relapsed B-ALL patients after allo-HSCT include chemotherapy, donor lymphocyte infusion (DLI), and a second allo-HSCT. In all these salvage treatments, a low rate of remission and short survival has been evidenced (2). Also, therapies such as DLI and the second allo-HSCT can cause acute graft-versus-host disease (aGVHD), a severe and lethal immune response (3). Effective new treatment strategies for relapsed patients after allo-HSCT are urgently needed.

Anti-CD19 chimeric antigen receptor modified (anti-CD19-CAR) T cell therapy has been effective eliminating malignant B cells (4). It has a high remission rate and long-term remission in patients with relapsed/refractory (R/R) B-ALL (5–7). Although this therapy is effective, it can cause severe toxicities. These toxicities include the cytokine release syndrome (CRS) and CAR-T cell-related encephalopathy syndrome (CRES), which are severe or even fatal (8). CRS is a complication associated with significantly elevated serum levels of inflammatory cytokines (8, 9).

Some studies have reported that donor-derived allogeneic anti-CD19-CAR T-cell therapy can effectively eradicate B-cell malignancies in B-ALL patients relapsing after allo-HSCT (10, 11). Allogeneic anti-CD19-CAR T-cell therapy can achieve a surprisingly good result without serious aGVHD in most of these research (11, 12).

In our clinical trial, we did not observe the mild aGVHD after anti-CD19-CAR T-cell therapy found in other studies. The anti-CD19-CAR T-cells used in this study were humanized anti-CD19-CAR T cells. It has been reported that patients can produce an immune response specific to murine scFv after murine CD19-CAR-T therapy, and exhibit a subsequent failure to murine CD19-CAR-T treatment (13). Taking advantage of humanized scFvs might reduce the immunogenicity of murine CD19 CAR-T cells and improve the longevity of anti-CD19-CAR T cells in patients (14). After the humanized anti-CD19-CAR T-cell therapy, the development of aGVHD was observed in 10 patients (66.67%) of all 15 B-ALL patients who relapsed after allo-HSCT. Four patients developed grade III-IV aGVHD. The donors of these four patients were all haploid donors. However, all the patients who survived to date experienced aGVHD after humanized anti-CD19-CAR T cell therapy.

## Patients and Methods

### Participants in the Clinical Trial

Fifteen B-cell acute lymphoblastic leukemia (ALL) patients who relapsed after allogeneic hematopoietic stem cell transplant (allo-HSCT) between April 2018 and February 2020 were admitted to the Department of Hematology in Tianjin First Center Hospital (Tianjin, China). They all had high CD19 expression in malignant B cells analyzed by flow cytometry (FCM). They were enrolled in clinical trials of anti-CD19 chimeric antigen receptor modified (anti-CD19-CAR) T cell expressing humanized anti-CD19 scFv and 4-1BB-CD3ζ costimulatory-activation domains therapy *(ChiCTR1800019622 and ChiCTR1800019622)*. All patients provided informed consent before enrollment. The final follow-up visit for endpoint analysis was conducted on May 31, 2020.

### The Source of T-Cells for Anti-CD19-CAR T-Cell Therapy

Because the donor chimerism in peripheral blood in most patients was below 60%, the donors of allo-HSCT provided peripheral blood mononuclear cells (PBMCs) for this anti-CD19-CAR T-cell therapy.

### Generation and Detection of Anti-CD19-CAR T Cells and Transduction Efficiency

PBMCs were collected and isolated by Ficoll density gradient centrifugation. CD3+ T cells were selected by CD3 microbeads (Miltenyi Biotec, Inc., Cambridge, MA, USA), stimulated by anti-CD3/anti-CD28 mAb-coated Human T-Expander beads (Cat. no. 11141D; Thermo Fisher Scientific, Inc., Waltham, MA, USA) and cultured in T-cell medium X-Vivo 15 (Lonza Group, Ltd., Basel, Switzerland) supplemented with 250 IU/mL interleukin-2 (IL-2; Proleukin; Novartis International AG, Basel, Switzerland). All the CD3+ T cells (3 × 10^6^) were transduced with a lentiviral vector encoding humanized CD19 CAR constructs (10 μg, lenti-CD19-2rd-CAR; Shanghai Genbase Biotechnology Co., Ltd., Shanghai, CHINA) and cultured in media containing recombinant human IL-2 (250 U/ml). On the 12th day of cultivation, transduction efficiencies of anti-CD19-CAR were analyzed by flow cytometry (FCM) (BD Biosciences, San Jose, CA, USA).

### The lympho-Depleting Chemotherapy and Anti-CD19-CAR T-Cell Infusion

All patients received lympho-depleting chemotherapy with fludarabine (30 mg/m^2^) and cyclophosphamide (400 mg/m^2^) from day −4 until day −2. The humanized anti-CD19-CAR T-cells derived from their donors were infused (1 × 10^6^ cells/kg) on day 0.

### Therapeutic Effect Observation and Donor Chimerism Analysis

Therapy responses and minimal residual disease (MRD) analysis were assessed from day 14 post infusion. Disease status was defined as a complete response (CR), CR with incomplete count recovery (CRi), no remission (NR). Leukemia free survival (LFS) after the anti-CD19-CAR T-cell therapy was calculated from the date of CR to the date of disease recurrence. The changes in donor chimerism in bone marrow (BM) were analyzed using fluorescent-labeled multiple PCR amplification of short-tandem repeats (STR) analysis at the same time.

Disease status was defined as complete response (CR), CR with incomplete count recovery (Cri), or no remission (NR).

### Acute Graft-Versus-Host Disease (aGVHD) After Anti-CD19-CAR T-Cell Therapy

The occurrence and extent of aGVHD were observed in the 15 patients from the day of anti-CD19-CAR T-cell infusion to the disappearance of aGVHD or death. Acute and chronic GVHD were classified using the Glucksberg and Seattle classical scales, respectively (15, 16).

### Adverse Events and Anti-CD19-CAR T-Cell Expansion

The side effects were observed from 0 to 14 days after CAR T-cell infusion. The cytokine release syndrome (CRS) was graded according to the adopted CRS scoring system and the National Cancer Institute Common Terminology Criteria for Adverse Events v4.03 after CAR T-cell infusion (17).

The secretion levels of cytokines, including interleukin-6 (IL-6), IL-2R, tumor necrosis factor-α (TNF-α), and IL-8 were measured on days 0, 7, 14, 21, and 28 by enzyme linked immunosorbent assay with a double-antibody one-step sandwich method. The proportions of CAR-T cells and the DNA level of the anti-CD19-CAR gene were detected around 110 days after anti-CD19-CAR T-cell infusion. The expansion of anti-CD19-CAR T-cells in CD3+ T cells in peripheral blood was observed using FCM. The DNA levels of the anti-CD19-CAR gene were detected by quantitative polymerase chain reaction (qPCR).

### Follow-Up

A follow-up was done starting from the date of anti-CD19-CAR T-cell infusion until the patients died. LFS was calculated from the date of CR/CRi after anti-CD19-CAR T-cell therapy to the date of relapse. OS was calculated from the date of CR after anti-CD19-CAR T-cell therapy to the date of death from any cause.

### Statistical Analysis

Data were expressed as mean ± SE. CRS was grouped and compared by Mann-Whitney rank and inspection. Non-normal distribution data are expressed as the median and interquartile range (IQR). Pearson correlation coefficient was used for evaluating the correlation between different factors. The LFS and OS probabilities were estimated with the Kaplan-Meier method and were compared using the log-rank test. All statistical analyses were computed using SPSS (version 17.0). *P* < 0.05 were considered significant.

## Results

### Characteristics of the Patients in Our Study

All patients enrolled in our study were B-ALL patients who relapsed after allo-HSCT. Reviewing their medical history, revealed that four patients (Pt 6#, 8#, 10#, and 13#) received chemotherapy after recurrence and before anti-CD19-CAR T-cell therapy. The detailed characteristics of all patients are shown in Table 1. The median proportion of leukemia cells was 43.73% (IQR 5.6–82.0) in BM and 30.01% (IQR 2.6–66.8) in peripheral blood (PB) when they were enrolled. The median proportion of donor chimerism in BM was 48.77% (IQR 8.82–85.16) when they were enrolled. The median time from relapse to CAR-T therapy was 1.27 (IQR 0.5–3.0) months. All patients had no GVHD when they enrolled in this clinical trial.

**Table 1:** Patients baseline and therapy-related characteristics.

**Patient**	**Age**	**Diagnosis**	**Donor Type**	**GVHD before relapse**	**Time from transplant to relapse**	**Therapy after relapse**	**Blasts in BM before CAR-T Therapy (%)**	**Blasts in PB before CAR-T Therapy (%)**	**Donor chimerism (BM) (%)**	**Time from relapse to CAR-T therapy**	**Time from stop immuno-suppressive therapy to CAR-T therapy**
Pt1#	41–45	B-ALL (Ph+)	MMUDT (8/10)	Grade I aGVHD, cGVHD	58 months	TKI	60.4	36.5	8.82	0.5 months	51 months
Pt 2#	46–50	B- ALL	MSDT	Grade II aGVHD, cGVHD	18 months	No	26.2	16.4	20.55	0.5 months	7 months
Pt 3#	56–60	B-ALL	Haplo-HSCT (7/10)	Grade I aGVHD	11 months	No	5.6	2.6	79.12	1 months	9 months
Pt 4#	31–35	B-ALL	Haplo-HSCT (5/10)	No	5 months	No	68.8	46.5	39.35	0.5 months	3 months
Pt 5#	21–25	B-ALL	Haplo-HSCT (5/10)	Grade I aGVHD, cGVHD	15 months	No	45.2	22.9	48.36	0.5 months	6 months
Pt 6#	16–20	B-ALL	MUDT	Grade II aGVHD	4 months	Two courses	58.4	39.4	23.12	2 months	3 months
Pt 7#	11–15	B-ALL (Ph+)	Haplo-HSCT (5/10)	No	6 months	No	16.2	8.6	83.02	1 months	5 months
Pt 8#	11–15	B-ALL	MSDT	Grade I aGVHD	14 months	One course	32.8	16.8	77.13	1 months	9 months
Pt 9#	21–25	B-ALL (Ph+)	Haplo-HSCT (5/10)	No	13 months	TKI	48.6	33.5	36.18	0.5 months	11 months
Pt 10#	56–60	B-ALL	MSDT	Grade I aGVHD	9 months	Two courses	52.4	50.4	52.08	3 months	4 months
Pt 11#	21–25	B-ALL	MSDT	No	24 months	No	40.8	29.8	50.25	0.5 months	20 months
Pt 12#	16–20	B-ALL (Ph+)	Haplo-HSCT (5/10)	Grade I aGVHD	9 months	TKI	82.0	66.8	29.14	1 months	4 months
Pt 13#	21–25	B-ALL	Haplo-HSCT (5/10)	Grade II aGVHD	15 months	Three courses	12.8	8.8	85.16	6 months	6 months
Pt 14#	16–20	B-ALL	Haplo-HSCT (5/10)	Grade II aGVHD	7 months	No	40.2	25.5	56.75	0.5 months	3 months
Pt 15#	11–15	B-ALL	Haplo-HSCT (5/10)	Grade I aGVHD	5 months	No	65.5	45.6	42.47	0.5 months	0.5 months

*HLA-matched sibling donor transplantation, MSDT; HLA-matched unrelated donor transplantation, MUDT; HLA-mismatched unrelated donor transplantation, MMUDT; Haploid donor transplantation, Haplo-HSCT; Tyrosine kinase inhibitor, TKI*.

*Chemotherapy after relapse: Pt 6# Two courses of VDCP. Pt 8# One course of VCP. Pt 10# One course of FLAG and one course of VDCP*.

*Pt 13# Two courses of Hyper-CVAD and one course of FLAG*.

### The Transduction Efficiency and Immune Phenotype of Patients' T Lymphocytes

The expression of CD3+ T cells in the culture system was 99.2% after screening with CD3 microbeads. The mean transduction efficiency of the anti-CD19-CAR was 55.27 ± 12.38%. The mean humanized anti-CD19-CAR T-cells of all patients infused in this therapy was 1.26 ± 0.36 × 10^6^ cells/kg. The mean CD3+ T cells infused in this therapy was 2.28 ± 0.61 × 10^6^ cells/kg.

### Therapeutic Effect Observation and Donor Chimerism Analysis

Fourteen days post anti-CD19-CAR T-cell infusion, 13 patients (13/15, 86.67%) achieved CR/CRi, six patients (6/15, 40.0%) achieved CR, seven patients (7/15, 46.67%) achieved CRi, and 11 patients (11/15, 73.33%) achieved MRD negative status. The Pt 6# was evaluated as NR after this therapy. Because of the extramedullary leukemia of Pt 13#, he did not achieve CR/CRi, even though there were no leukemia cells in his BM. Donor chimerism in BM rose to 99.81 ± 0.20% in the 13 patients and achieved CR/Cri at 14 days after anti-CD19-CAR T-cell infusion. However, donor chimerism in BM of Pt 6# was only 10.72%, while donor chimerism in BM of Pt 13# was 99.56% (Table 2).

**Table 2:** The therapeutic effect, donor chimerism analysis, and acute GVHD after anti-CD19-CAR T-cell therapy.

**Patient**	**Donor Type**	**CR/Cri**	**MRD**	**Donor chimerism (%)**	**Time of aGVHD (Days after CAR-T therapy)**	**Grade of aGVHD**	**Site of aGVHD**	**Therapy to aGVHD**
Pt 1#	MMUDT (8/10)	Cri	Negative	99.86	60–102	II	S2, G1	Glucocorticoid, CsA
Pt 2#	MSDT	CR	Negative	99.83	35–97	I	S2	Glucocorticoid
Pt 3#	Haplo-HSCT (7/10)	CR	Negative	99.92	32–77	II	G1	Glucocorticoid
Pt 4#	Haplo-HSCT (5/10)	Cri	Negative	99.86	52–64	III	L3	Glucocorticoid, CsA, Anti-CD25 monoclonal antibody
Pt 5#	Haplo-HSCT (5/10)	Cri	Negative	99.91	24–104	IV	S3, L2, G4	Glucocorticoid, CsA, Ruxolitinib
Pt 6#	MUDT	NR	Positive	10.72	0	0	-	-
Pt 7#	Haplo-HSCT (5/10)	CR	Negative	99.94	0	0	-	-
Pt 8#	MSDT	CR	Positive	99.94	51–93	I	S1	Glucocorticoid
Pt 9#	Haplo-HSCT (5/10)	CR	Negative	99.94	0	0	-	-
Pt 10#	MSDT	Cri	Positive	99.77	0	0	-	-
Pt 11#	MSDT	CR	Negative	99.66	42–74	I	S1	Glucocorticoid
Pt 12#	Haplo-HSCT (5/10)	Cri	Negative	99.91	0	0	-	-
Pt 13#	Haplo-HSCT (5/10)	NR	Positive	99.56	31–102	III	S3, G3	Glucocorticoid, CsA, Ruxolitinib
Pt 14#	Haplo-HSCT (5/10)	Cri	Negative	99.86	21–73	IV	S4, L2, G4	Glucocorticoid, CsA, Ruxolitinib
Pt 15#	Haplo-HSCT (5/10)	Cri	Negative	99.17	14–56	I	S1	Glucocorticoid

*S, Skin; L, Liver, G, Gastrointestinal*.

### Acute GVHD After Anti-CD19-CAR T-Cell Therapy

The development of aGVHD was observed in ten patients (10/15, 66.67%) in this group of B-ALL patients relapsed after allo-HSCT. The donor type of these ten patients were haploid donors (Pt 3#, 4#, 5#, 13#, 14#, 15#), HLA-matched sibling donors (Pt 2#, 8#, 11#), and HLA-mismatched unrelated donor (Pt 1#). Six patients (Pt 1#, 2#, 3#, 8#, 11#, and 15#) (6/15, 40.0%) developed grade I-II of aGVHD from 14 days to 60 days post anti-CD19-CAR T-cell infusion. Four patients (Pt 4#, 5#, 13#, and 14#) (4/15, 26.67%) developed grade III-IV aGVHD from 21 days to 52 days post anti-CD19-CAR T-cell infusion. The other five patients (5/15, 33.33%) did not develop aGVHD. Donor selection, aGVHD occurrence and duration are shown in Table 2. Patients with grade I-II of aGVHD were controlled by glucocorticoid and cyclosporin A (CsA) therapy. Patients with grade III-IV of aGVHD were controlled by glucocorticoid, CsA, anti-CD25 monoclonal antibody and JAK1/JAK2 kinase inhibitor (Ruxolitinib) therapy. There were no aGVHD related deaths in our study.

### Adverse Events Observation of Anti-CD19-CAR T-Cell Therapy

After the CAR-T cell infusion, patients manifested pyrexia with chills, accompanied by muscular weakness, fatigue, tachycardia, nausea, decreased appetite, increased serum alanine transaminase(ALT), glutamic oxalacetic transaminase(AST) and bilirubin, acute kidney injury and oliguria, electrolyte disturbance, and hematological toxicity (Table 3). Notable adverse events (AEs) in all 15 patients were grade 1–2 cytokine release syndrome (CRS) in ten patients (10/15, 66.67%) and grade 3–4 CRS in five patients (5/15, 33.33%). No patients were diagnosed with CAR-T cell-related encephalopathy syndrome (CRES) in all the process of the treatment. No CRS or CRES related deaths occurred in our study. Fourteen of the 15 patients (14/15, 93.33%) developed grade 3–4 hematological toxicity post anti-CD19-CAR T-cell infusion. The other patient developed grade 2 hematological toxicity only (Table 3).

**Table 3:** Adverse events observation of anti-CD19-CAR T-cell therapy.

**Events**	**The incidence**
**General condition**	
Temperature ≥38°C (fever)	13/15 (86.67%)
Chills	6/15 (40.00%)
Muscular weakness	10/15 (66.67%)
Rash	2/15 (13.33%)
Systolic blood pressure <90 mm Hg (hypotension)	1/15 (6.67%)
Needing oxygen for SaO_2_ >90% (hypoxia)	2/15 (13.33%)
Fatigue	11/15 (73.33 %)
Weight loss	4/15 (26.67%)
**Organ toxicities**	
**Cardiac**	
Tachycardia	6/15 (40.00%)
Arrhythmias	1/15 (6.67%)
Heart block	0/15 (0%)
**Respiratory**	
Hypoxia	3/15 (20.00%)
Dyspnea	2/15 (13.33%)
Cough	5/15 (33.33%)
Pleural effusion	2/15 (13.33%)
**Gastrointestinal**	
Nausea	7/15 (46.67%)
Vomiting	3/15 (20.00%)
Decreased appetite	8/15 (53.33%)
**Hepatic**	
Increased serum ALT, AST	8/15 (53.33%)
Increased serum bilirubin levels	5/15 (33.33%)
**Renal**	
Acute kidney injury (increased serum creatinine levels)	6/15 (40.00%)
Oliguria	5/15 (33.33%)
**The electrolyte**	
Hypokalaemia	7/15 (46.67%)
Hypocalcaemia	7/15 (46.67%)
Hyperglycaemia	4/15 (26.67%)
**Coagulopathy**	
Disseminated intravascular coagulation	1/15 (6.67%)
**Neurological**	
Encephalopathy	0/15 (0%)
Confused state	0/15 (0%)
Dizziness	2/15 (13.33%)
Aphasia	0/15 (0%)
Somnolence	3/15 (20.00%)
**Hematological**	
Neutropenia (grade 3/4) (<1*10^9^/*L*)	14/15 (93.33%)
Anemia (grade 3/4) (<80 g/L)	12/15 (80.00%)
Thrombocytopenia (grade 3/4) (<50*10^9^/*L*)	14/15 (93.33%)
**Cytokine release syndrome (CRS)**	
Grade 0 CRS	0/15 (0%)
Grade 1 CRS	5/15 (33.33%)
Grade 2 CRS	5/15 (33.33%)
Grade 3 CRS	3/15 (20.00%)
Grade 4 CRS	2/15 (13.33%)
Grade 5 CRS	0/15(0%)

**Figure 1:**
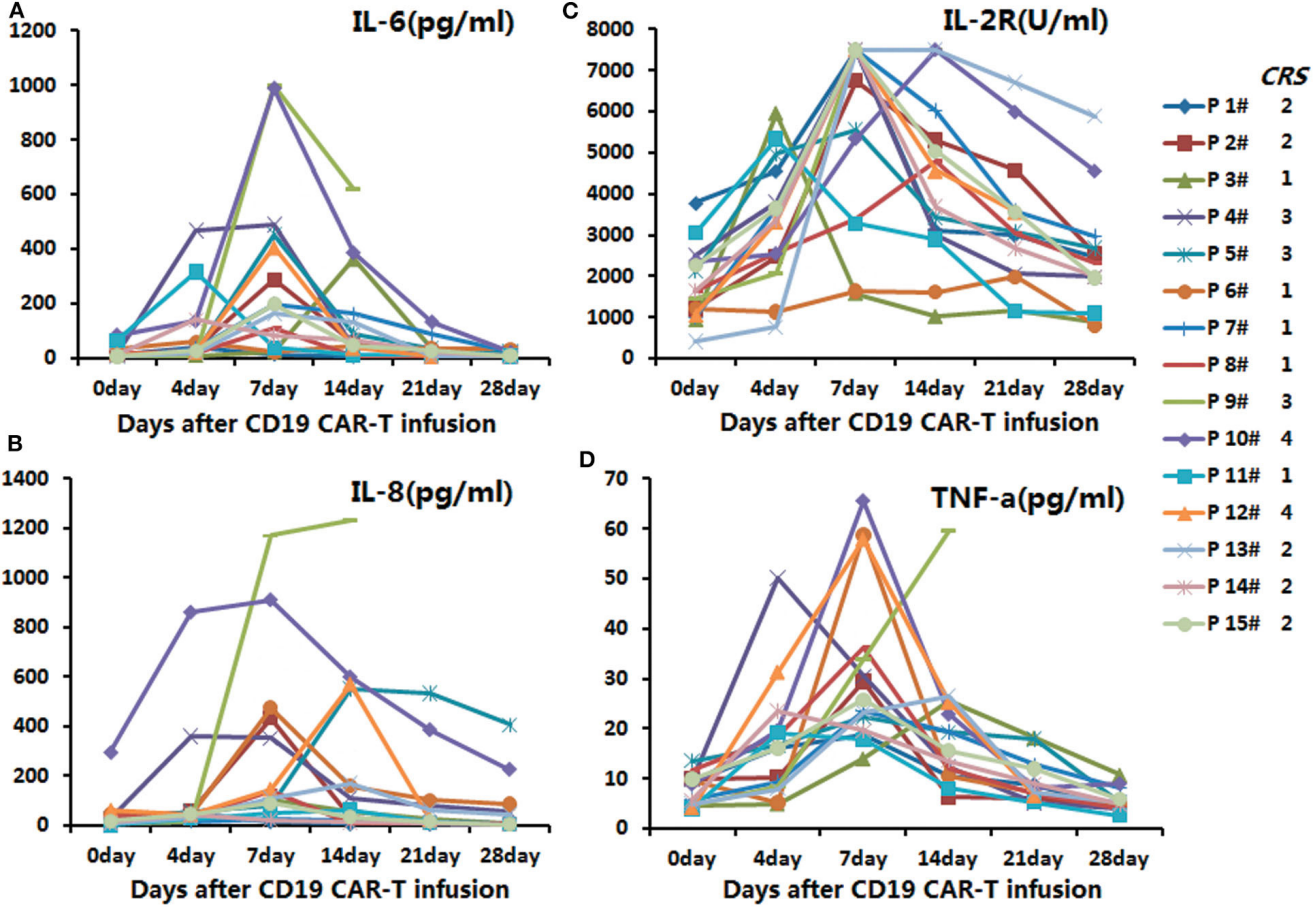
The secretion levels of IL-6, IL-2R, TNF-α, and IL-8 in the anti-CD19-CAR T-cell therapy. The peaks of the cytokines were on days 4–7 post infusion of anti-CD19-CAR T-cells, and the levels then declined 14 days post infusion. **(A)** The secretion levels of IL-6 in the anti-CD19-CAR T-cell therapy. **(B)** The secretion levels of IL-8 in the anti-CD19-CAR T-cell therapy. **(C)** The secretion levels of IL-2R in the anti-CD19-CAR T-cell therapy. **(D)** The secretion levels of TNF-a in the anti-CD19-CAR T-cell therapy.

In the anti-CD19-CAR T-cell therapy, the cytokines' peaks occurred on days four to seven post infusion of anti-CD19-CAR T-cells, and declined 14 days after the infusion (Figure 1).

All patients were given antipyretic drugs and methylprednisolone to overcome the AEs. Only the patients 10# and 12#, who developed grade 4 CRS were given tocilizumab during therapy. The AEs of the other patients were relieved 9–16 days post infusion.

### Expansion of Anti-CD19-CAR T-Cells and Anti-CD19-CAR Gene Expression

**Figure 2:**
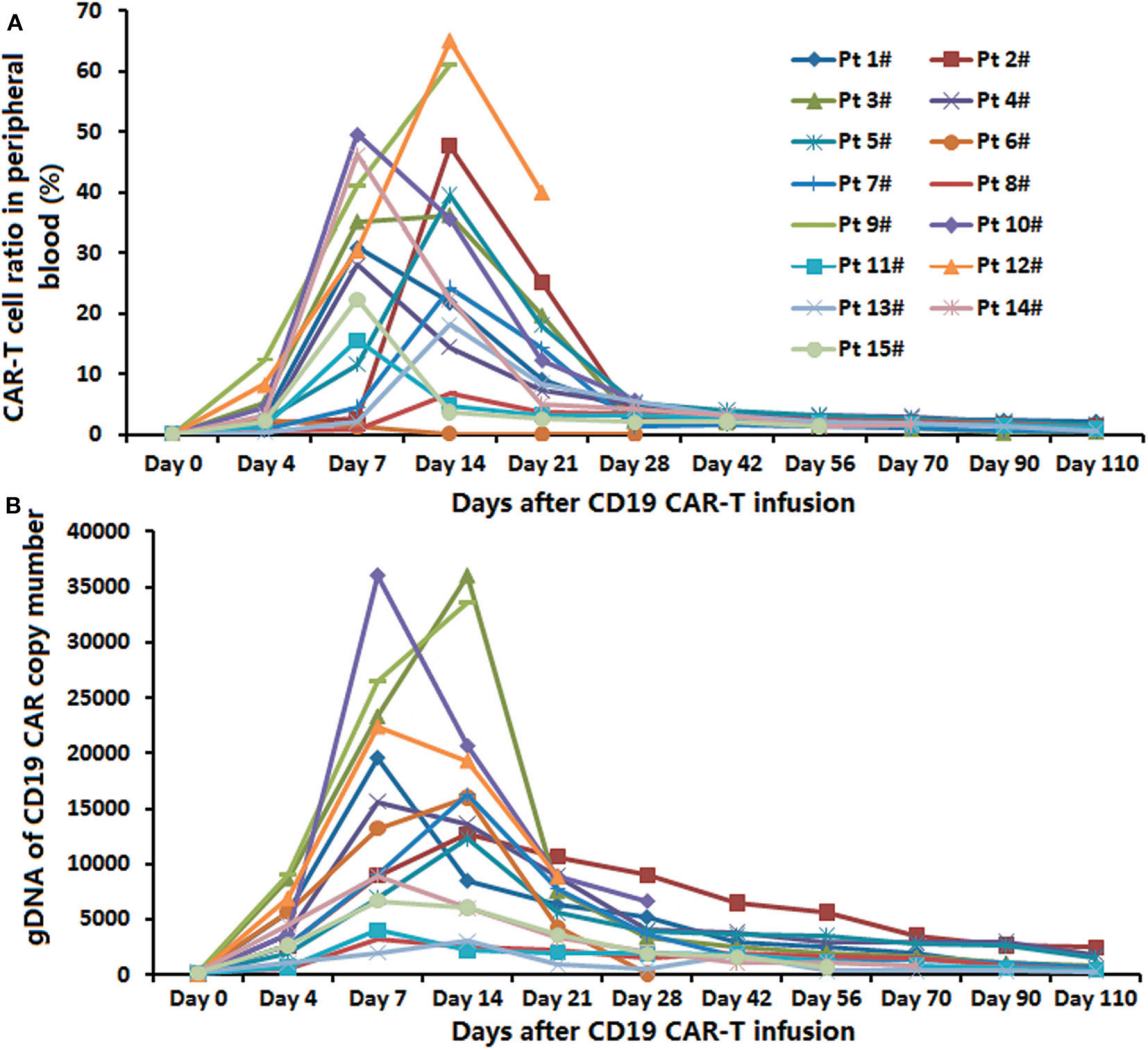
Expansion of anti-CD19-CAR T-cells and anti-CD19-CAR gene. **(A)** The average expansion peak of anti-CD19-CAR T cells was 32.54 ± 19.94% on days 4–14 (median time was 11.31 ± 3.47 days) after the infusion. The mean level of anti-CD19-CAR T cells was 2.58 ± 1.32% when the patients developed aGVHD. **(B)** DNA level of anti-CD19-CAR gene showed the same trend.

The peak proportions of CAR-T cells in CD3+ T cells in peripheral blood were detected in the process of the anti-CD19-CAR T cell therapy. The average expansion peak of anti-CD19-CAR T cells was 32.92 ± 20.44% on days 4 to 14(median time was 11.31 ± 3.47 days) after the infusion (Figure 2A). The mean level of anti-CD19-CAR T cells was 1.98 ± 1.54% when the patients developed aGVHD. We did not observe the expansion peak of anti-CD19-CAR T cells or anti-CD19-CAR gene expression again in the process of aGVHD. The DNA levels of anti-CD19-CAR genes showed the same trend (Figure 2B).

### Correlation Analysis Between the aGVHD and Cytokine Levels, CAR-T Cell Levels, and T Cell Subsets

**Figure 3:**
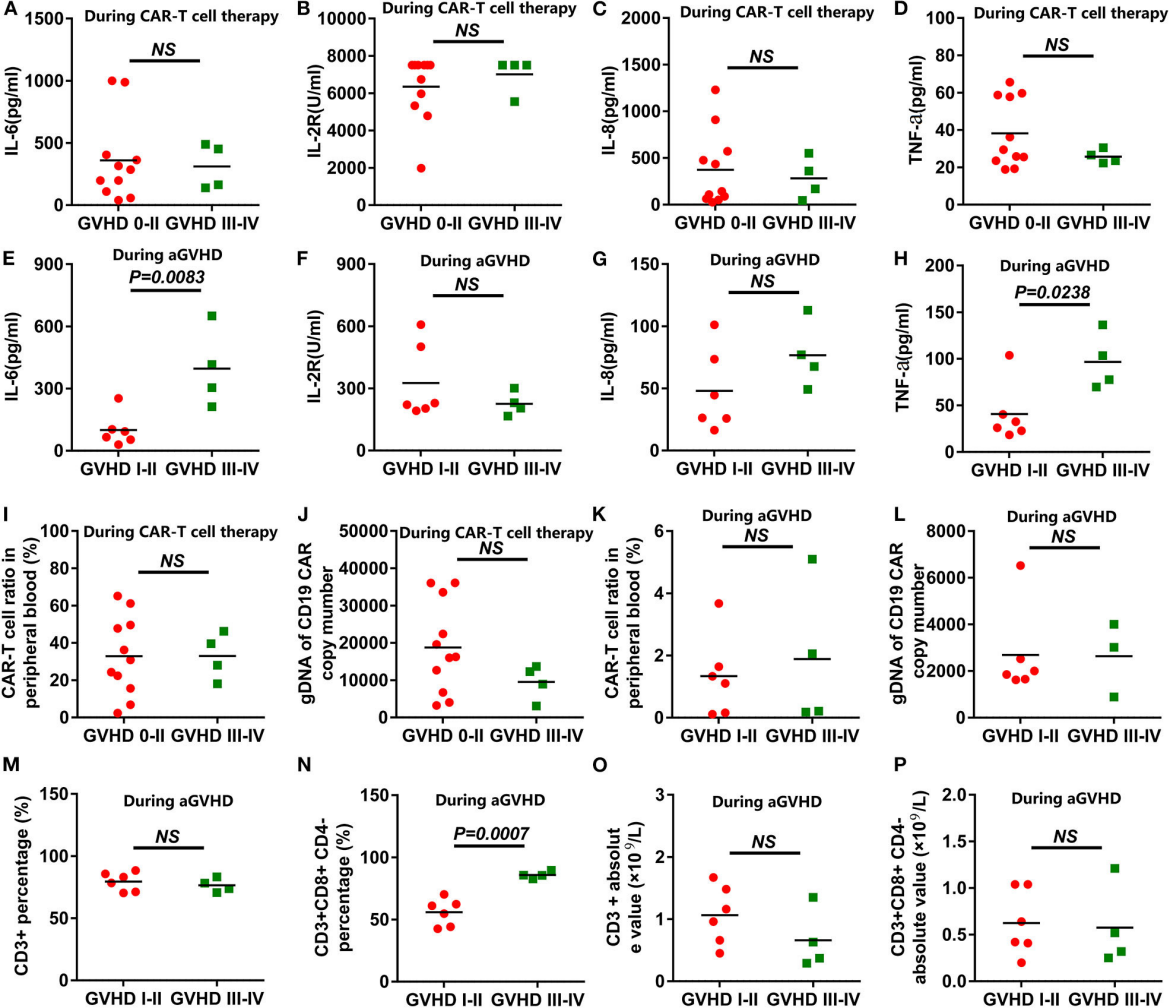
Correlation analysis between the aGVHD and the cytokine levels or CAR-T cell levels. **(A–D)** The peaks of the cytokines in the anti-CD19-CAR T-cell therapy were uncorrelated with aGVHD levels. **(E,H)** The peaks of IL-6 and TNF-a in grade I-II in the aGVHD group were lower than those of the grade III-IV in the aGVHD group during the occurrence of aGVHD. **(F,G)** The peaks of the IL-2R and IL-8 during the occurrence of aGVHD were uncorrelated with aGVHD levels. **(I–L)** The peaks of anti-CD19-CAR T cells and anti-CD19-CAR gene were uncorrelated with aGVHD levels during the anti-CD19-CAR T-cell therapy and the occurrence of aGVHD. **(N)** The CD3+CD8+CD4- percentages in grade I-II in the aGVHD group were lower than that of the grade III-IV in the aGVHD group. **(M,O,P)** The CD3+ percentage, the CD3+ absolute value and the CD3+CD8+CD4- absolute value were uncorrelated with aGVHD levels during the occurrence of aGVHD.

All the cytokines' peaks occurred before 14 days post CAR-T cell infusion, while the AEs of the patients had relieved 16 days post infusion, and all aGVHD cases occurred after 21 days post infusion. The 15 patients were divided into grade 0-II of aGVHD and grade III-IV of aGVHD groups. The cytokine levels in the peripheral blood of patients receiving anti-CD19-CAR T-cell therapy were uncorrelated with aGVHD levels (Figures 3A–D). During the occurrence of aGVHD, the peaks of IL-6 and TNF-a in grade I-II of aGVHD were lower than in grade III-IV of aGVHD (Figures 3E,H). The peaks of IL-2R and IL-8 during the occurrence of aGVHD were uncorrelated with aGVHD levels (Figures 3F,G). In all patients, the peaks of anti-CD19-CAR T cells and anti-CD19-CAR gene expression in the peripheral blood were uncorrelated with aGVHD levels (Figures 3I,J). The anti-CD19-CAR T cells and anti-CD19-CAR gene expression changes during the occurrence of aGVHD were uncorrelated with aGVHD levels (Figures 3K,L). The CD3+CD8+CD4- percentages in peripheral blood in grade I-II of aGVHD patients were lower than those of grade III-IV of aGVHD patients (Figure 3N). However,CD3+ percentage, CD3+ absolute value, and CD3+CD8+CD4- absolute value during the occurrence of aGVHD were uncorrelated with the aGVHD levels (Figures 3M–P).

### Follow-Up

**Figure 4:**
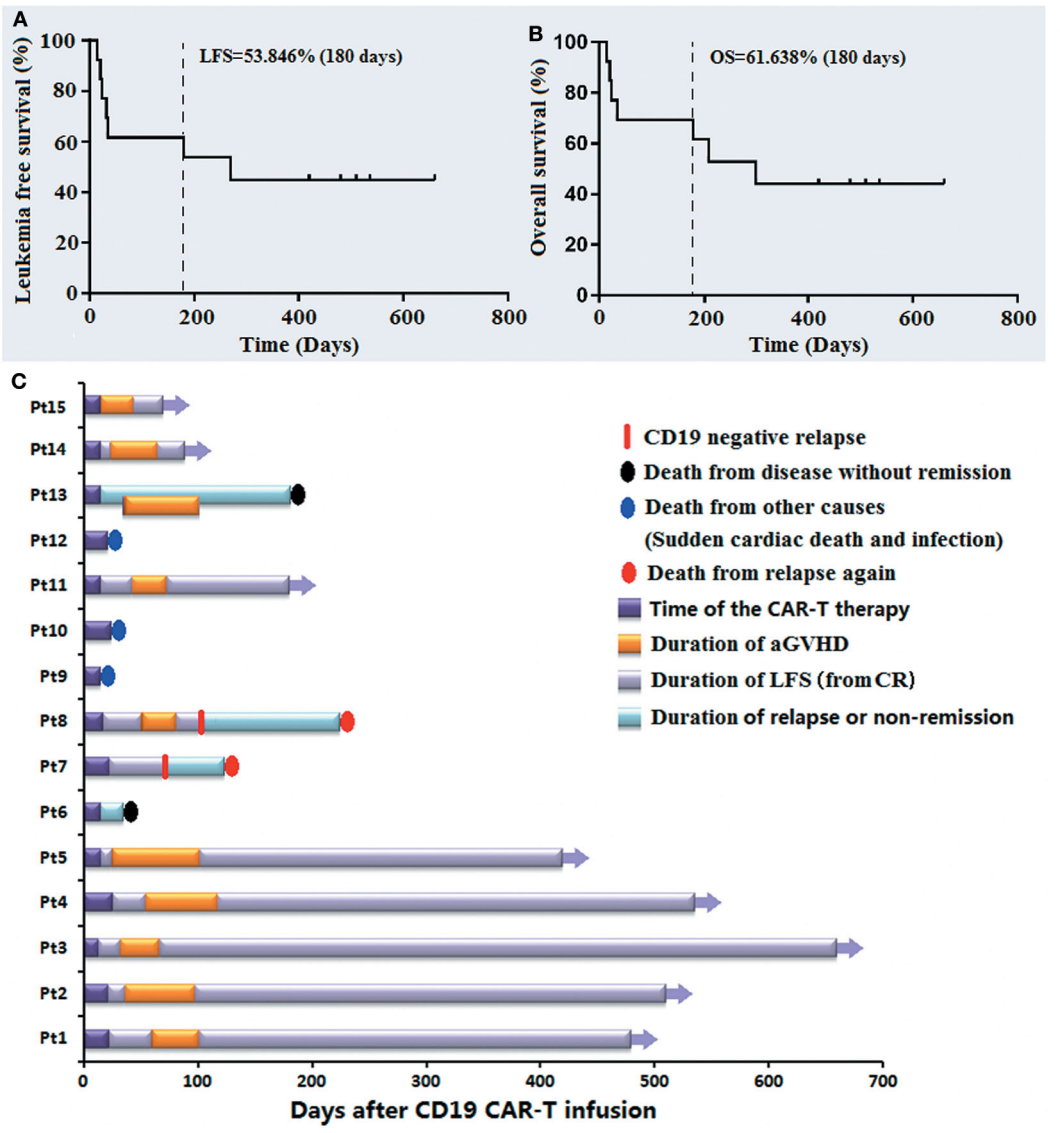
The follow-up post anti-CD19-CAR-T therapy of the B-ALL patients who relapsed after allo-HSCT. **(A,B)** The LFS and OS of the 13 patients on 180 days were 53.846 and 61.638%. **(C)** The LFS, OS, occurrence of aGVHD, cause of death and CD19 expression at the moment of recurrence are listed.

The median follow-up time of the 15 patients was 258.69 days (15–660 days). By May 31, 2020, the rates of LFS and OS during 180 days were 53.846 and 61.638%, respectively (Figures 4A,B). Pt 9# died of sudden cardiac death 15 days post infusion, Pt 10# and 12# died of serious infection at 21 and 24 days post infusion, respectively, when they had achieved CRi and were relieved from the AEs of this therapy. Pt 6# and Pt 13#, who had not achieved CR/CRi from this therapy died of disease progression 35 and 181 days post infusion, respectively. The Pt 7# and Pt 8# developed recurrence again at 75 and 103 days after the anti-CD19-CAR T cell infusion, respectively, and died 125 and 227 days after infusion, respectively. Figure 4 shows the LFS, OS, the occurrence of aGVHD, the cause of death and the CD19 expression at the monent of recurrence up to January 31, 2020. All patients who survived experienced aGVHD after anti-CD19-CAR T cell therapy. In particular, the LFS of Pt 1# to Pt 5# and Pt 11# was 480, 512, 660, 536, 421, and 183 days, respectively.

## Discussion

Allo-HSCT therapy is an effective treatment to improve the survival of B-ALL patients. However, several B-ALL patients with high-risk factors are still at a high risk of relapse after allo-HSCT and poor long-term survival (18–20). Recurrence after allo-HSCT was the main cause of death after allo-HSCT (21). The B-ALL patients relapsed after allo-HSCT had a median survival of only 5.5 months (1). DLI with or without chemotherapy, is essential therapy to B-ALL patients who relapsed after allo-HSCT. However, Only some patients responded to DLI therapy, and these patients had an increased risk of aGVHD which can be caused by the infusion of a large number of donor T cells (22–24). Thus, a 20% treatment-related mortality or the lack of response to it resulted in a median survival period of 6 months for the B-ALL patients that relapsed after allo-HSCT (3).

Anti-CD19-CAR T-cell therapy had significant clinical efficacy in relapsed/refractory B-ALL patients who had not received allo-HSCT (25). An early study focused on donor anti-CD19-CAR T-cell therapy for such patients (10). The anti-CD19-CAR T-cell therapy derived from donor T cells was a selection of salvage therapy to B-ALL patients who relapsed after allo-HSCT, because allogeneic anti-CD19-CAR T-cells exerted a graft versus leukemia(GVL) effect (11, 26–28). In these studies, donor anti-CD19-CAR T-cell therapy resulted in a high remission rate compared with conventional therapeutic approaches such as DLI. Two reviews summarized the effects and side effects of this therapy (29, 30). It was effective for minimal residual disease without serious side-effects, including aGVHD, CRS, and CRES. Therefore, effective and low toxicity anti-CD19-CAR T-cell therapy can be an option to overcome the poor prognosis of B-ALL patients who relapsed after allo-HSCT. However, the long-term efficacy and survival time for this therapy are not mentioned sufficiently.

In our study, 13 patients (86.67%) achieved CR/CRi 14 days after the anti-CD19-CAR T-cell infusion, including six patients (40.0%) who achieved CR. Ten patients (66.67%) achieved CR 28 days after infusion. Ten patients (66.67%) developed grade 1-2 CRS, while five patients (33.33%) developed grade 3–4 CRS. The side-effects observed in our study were more serious than those in previous the studies (10, 11, 26–28). The high median proportion of leukemia cells in BM (43.73%) before enrollment can be one of the possible reasons for these serious side-effects. The development of aGVHD was observed in 10 patients (66.67%) in this group. Six patients developed grade I-II of aGVHD, while four patients developed grade III-IV of aGVHD. The grades of aGVHD in our study were higher than those in previous studies (10, 11, 26–28).

In previous studies, the reason for mild aGVHD after anti-CD19-CAR T-cell therapy was unclear. The risk of developing aGVHD after the anti-CD19-CAR T-cell infusion was different from the DLI infusion (31, 32). One explanation was that the dose of anti-CD19-CAR T-cell infusion was smaller than the dose of DLI. In our study, the mean anti-CD19-CAR T-cells infused in this therapy was 1.26 ± 0.36 × 10^6^ cells/kg and the mean CD3+ T cells infused was 2.28 ± 0.61 × 10^6^ cells/kg. Another explanation is that the persistence of anti-CD19-CAR T-cell was limited to <4 weeks in previous studies. In general, aGVHD occurred with a median of 4 weeks after the infusion of DLI. The mean level of anti-CD19-CAR T cells was 1.98 ± 1.54% when the patients developed aGVHD in this group of patients. The anti-CD19-CAR T cells were not eliminated from the patient at this time.

The anti-CD19-CAR T-cells used in our study were humanized anti-CD19-CAR T cells. HLA restricted T-cell mediated immune response to epitopes derived from murine scFv, which can affect the survival time of anti-CD19-CAR T-cells *in vivo* (33). Humanized anti-CD19-CAR T cells in our study can reduce the immunogenicity of murine CD19 CAR-T cells and prolong the survival time of cells in patients (34). Tumor burden was another critical factor that can influence the expansion of anti-CD19-CAR-T cells during this therapy (13, 35, 36). It can be another factor that contributes to the longer existential time of anti-CD19-CAR-T cells in our study. The last factor was that the donors of the four patients who developed grade III-IV of aGVHD were all haploid donors. Whether these factors are the reasons for the higher rate of aGVHD in this group of patients, needs to be expanded using more case-studies.

In our clinical trial, we did not observe mild aGVHD after the anti-CD19-CAR T-cell therapy in previous studies. However, the AEs and aGVHD in our study were serious but controllable. Patients who had an extended survival time developed aGVHD after this treatment. In particular, five patients had an LFS for more than 400 days after the anti-CD19-CAR T-cell therapy and subsequent aGVHD.

## Data Availability Statement

All datasets generated for this study are included in the article/supplementary material.

## Ethics Statement

The studies involving human participants were reviewed and approved by Tianjin First Center Hospital (Tianjin, China). The patients/participants provided their written informed consent to participate in this study.

## Author Contributions

QD and DY: conception and design and study supervision. PL: drafting or reviewing of the manuscript. ML, CL, WL, RC, QL, and NM: acquisition of data. JW: analysis and interpretation of data. All authors: writing and review of manuscript.

## Conflict of Interest

NM was employed by the company Shanghai Genbase Biotechnology Co., Ltd. The remaining authors declare that the research was conducted in the absence of any commercial or financial relationships that could be construed as a potential conflict of interest.
